# Spatiotemporal epidemiology of scarlet fever in Jiangsu Province, China, 2005–2015

**DOI:** 10.1186/s12879-017-2681-5

**Published:** 2017-08-30

**Authors:** Qi Zhang, Wendong Liu, Wang Ma, Yingying Shi, Ying Wu, Yuan Li, Shuyi Liang, Yefei Zhu, Minghao Zhou

**Affiliations:** 1 Department of Epidemiology and Biostatistics, School of Public Health, Nanjing Medical University, Nanjing, 211166 China; 2 Jiangsu Provincial Center for Disease Control and Prevention, Nanjing, 210009 China; 3 The Second Affiliated Hospital of Nanjing Medical University, Nanjing, 210011 China

**Keywords:** Scarlet fever, Spatiotemporal analysis, Epidemiology

## Abstract

**Background:**

A marked increase in the incidence rate of scarlet fever imposed a considerable burden on the health of children aged 5 to 15 years. The main purpose of this study was to depict the spatiotemporal epidemiological characteristics of scarlet fever in Jiangsu Province, China in order to develop and implement effective scientific prevention and control strategies.

**Methods:**

Smoothed map was used to demonstrate the spatial distribution of scarlet fever in Jiangsu Province. In addition, a retrospective space-time analysis based on a discrete Poisson model was utilized to detect clusters of scarlet fever from 2005 to 2015.

**Results:**

During the years 2005–2015, a total of 15,873 scarlet fever cases occurred in Jiangsu Province, with an average annual incidence rate of 1.87 per 100,000. A majority of the cases (83.67%) occurred in children aged 3 to 9 years. Each year, two seasonal incidence peaks were observed, the higher occurring between March and July, the lower between November and the following January. The incidence in the southern regions of the province was generally higher than that in the northern regions. Seven clusters, all of which occurred during incidence peaks, were detected via space-time scan statistical analysis. The most likely cluster and one of the secondary clusters were detected in the southern and northern high endemic regions, respectively.

**Conclusion:**

The prevalence of scarlet fever in Jiangsu Province had a marked seasonality variation and was relatively endemic in some regions. Children aged 3 to 9 years were the major victims of this disease, and kindergartens and primary schools were the focus of surveillance and control. Targeted strategies and measures should be taken to reduce the incidence.

## Background

Scarlet fever, an acute respiratory infectious disease caused by Group A *Streptococcus pyogenes* (GAS), most commonly occurs in winter and spring [1]. GAS usually spreads among the population via respiratory droplets or by direct contact with the mucus, saliva, or skin of an infected person. Some outbreaks of scarlet fever are even found to be food-borne [2–4]. Children from 5 to 15 years of age are vulnerable to scarlet fever [2, 5]. The signs and symptoms include sore throat, high fever, or skin infection, and the characteristic red rash that feels like sandpaper.

During the nineteenth century, scarlet fever, causing an enormous number of deaths, was a common fatal childhood infectious disease [6]. With improved nutrition and wide spread use of antibiotics, mortality markedly decreased and rarely occurred in the twentieth century [7, 8]. Scarlet fever is now a common mild contagious disease that with timely antibiotic therapy has a good prognosis. However, 3% to 5% of untreated cases can result in long-term complications, including rheumatic heart disease, hepatitis, and glomerulonephritis, that consume a large proportion of health care resources [9–11]. As a result of changes in prevalence strain, GAS antibiotic resistance, and the continued unavailability of an effective vaccine [12–17], scarlet fever outbreaks have been reported in many countries and regions, such as Vietnam, Australia, USA, China and England [2, 14, 17–20]. In recent years, the incidence of scarlet fever in China has experienced a sharp increase [1].

Advances in the geographic information system (GIS) and related technologies have fostered new opportunities to understand diseases from different perspectives. As a useful tool, GIS has been widely applied in controlling infectious diseases [21–23]. However, few studies have focused on the spatiotemporal characteristics of scarlet fever. Gehendra et al. conducted a spatial analysis of scarlet fever in Beijing and detected several high-risk areas. This was essential to take targeted efforts of prevention and control for this disease [24]. Zheng et al. also explored the clusters of the disease for Gansu Province [25]. In Jiangsu Province, some researchers described the epidemiology of scarlet fever, but none explored the spatiotemporal patterns [26–29].

In the present study, we concurrently used descriptive analysis and GIS methods to depict the spatial and temporal characteristics of scarlet fever in Jiangsu Province, China. The results might provide some clues for further epidemiological research and help scientists and health authorities to more effectively target their future surveillance and control efforts.

## Methods

### Study area

Jiangsu Province, located in latitude 30°46′–35°08′N and longitude 116°21′–121°54′E, is an eastern-coastal province of China. It consists of 13 cities, while each city consists of several counties. The province covers an area of 102,600 km^2^ surrounded by the Yellow Sea in the east, Zhejiang Province and Shanghai in the south, Anhui Province in the west, and Shandong Province in the north. The population in Jiangsu Province increased from 75 million to 80 million in the past decade.

### Data collection

The Nationwide Notifiable Infectious Diseases Reporting Information System (NIDRIS) was implemented in 2004, covering all health care institutions across China. Since then, notifiable infectious disease cases have been reported to this system in real time [30, 31].

The data of scarlet fever cases from 2005 to 2015 in Jiangsu Province were obtained from NIDRIS. The information about scarlet fever cases included sex, age, address, as well as date of onset. Demographic data were collected from the Data-center of China Public Health Science [32].

### Statistical analysis

Descriptive statistics were used to illustrate the characteristics of the population distribution and temporal distribution of scarlet fever. SAS software version 8.1 (SAS Institute Inc., Cary, NC, United States) was employed to investigate the level of statistical significance. A smoothed map of Ordinary Kriging [33] was produced in ArcGIS software version 10.0 (ESRI, Redlands, CA, USA) to indicate the spatial patterns of scarlet fever.

### Space-time scan statistic

SaTscan software version 9.4 [34], was utilized to detect clusters of scarlet fever in the study area from 2005 to 2015, using retrospective space-time analysis based on a discrete Poisson model [35, 36]. The space-time scan statistic is defined by a cylindrical window, with a circular geographic base and height corresponding to the time dimension. In this study, the spatial size of the scanning window was limited to 20% of the total population at risk and the length of time limited to 3 months. The null hypothesis of the analysis was that scarlet fever risk was not different inside and outside the window, while the alternative hypothesis was that the risk was elevated within the window compared to the outside. For each window, Log Likelihood Ratio (LLR) was calculated and a *p*-value (α = 0.05) estimated through Monte Carlo simulation. The window with the maximum LLR was defined as the most likely cluster, other windows with a statistically significant LLR were considered as secondary clusters.

## Results

### Demographic characteristics

A total of 15,873 cases of scarlet fever occurred during 2005–2015 in Jiangsu Province, with an average annual incidence rate of 1.87 per 100,000. Incidence in men was higher than that in women (χ^2^ = 968.58, *p* < 0.0001), with a male-to-female ratio of 1.66:1. The group aged 3 to 9 years accounted for 83.67% of all the reported cases and had the highest incidence rate of 22.06 per 100,000 (Table 1).

**Table 1:** Demographic characteristics of scarlet fever in Jiangsu Province, 2005–2015

Age group (years)	Male		Female		Total	
	Cases	Incidence	Cases	Incidence	Cases	Incidence
≤2	489	3.31	297	2.24	786	2.81
3–9	8292	25.99	4988	17.63	13,280	22.06
10–14	791	3.62	506	2.75	1297	3.22
≥15	325	0.09	185	0.05	510	0.07
Total	9897	2.31	5976	1.41	15,783	1.87

Note: incidence is the average annual incidence rate (per 100,000 populations)

### Temporal distribution

**Fig. 1:**
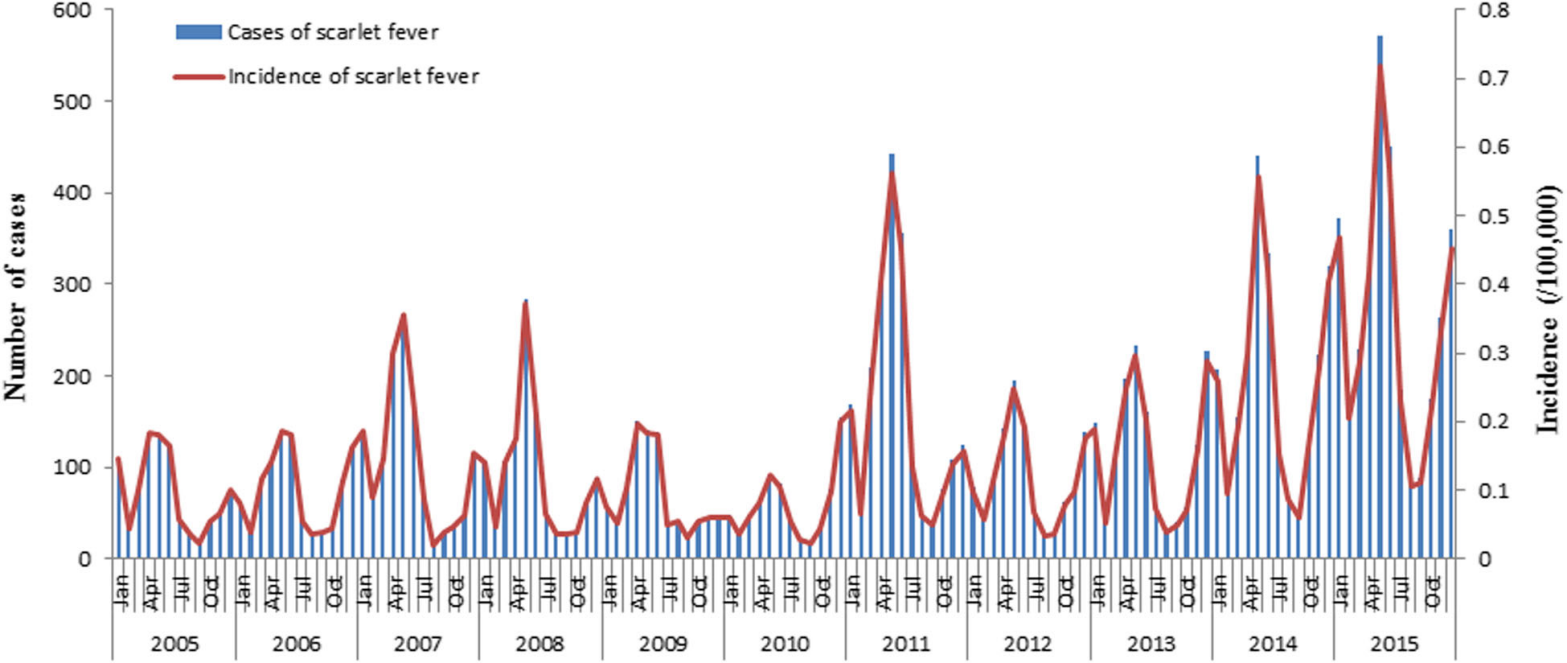
Monthly incidence and reported cases of scarlet fever in Jiangsu Province, 2005–2015

As shown in Fig. 1, there was an ascending long-term trend (χ^2^ = 2154.2, *p* < 0.0001) in the incidence of scarlet fever in the last 11 years in Jiangsu Province, and the highest annual incidence rate was in 2015 (4.08 per 100,000). Two incidence peaks were observed in each year, the higher between March and July, and the lower between November and the following January. Additionally, two incidence rate troughs were observed each year in August and February.

### Spatial distribution

**Fig. 2:**
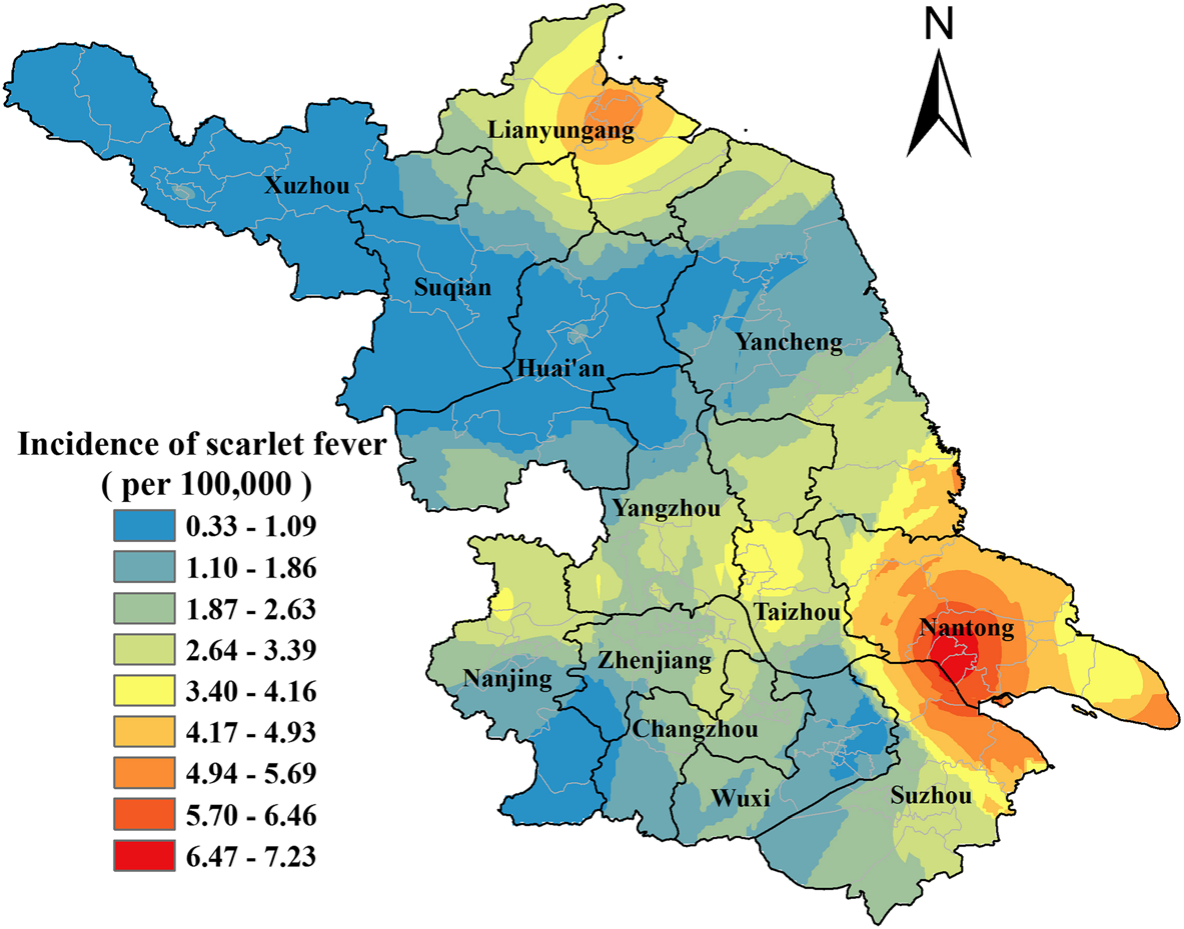
Smoothed map of incidence rate of scarlet fever in Jiangsu Province, 2005–2015

The southern regions in Jiangsu Province had a relatively high incidence of scarlet fever compared to the northern regions during 2005–2015. Two high endemic regions were observed, with one mainly located in Nantong city and Suzhou city and the other in Lianyungang city. Three northwest cities, namely Xuzhou, Suqian, and Huai’an were low endemic regions. The incidence rate ranged between 0.01 and 19.53 per 100,000 at the county level. Chongchuan district in Nantong city had the highest incidence rate of 7.23 per 100,000 populations (Fig. 2).

### Spatiotemporal clusters

**Fig. 3:**
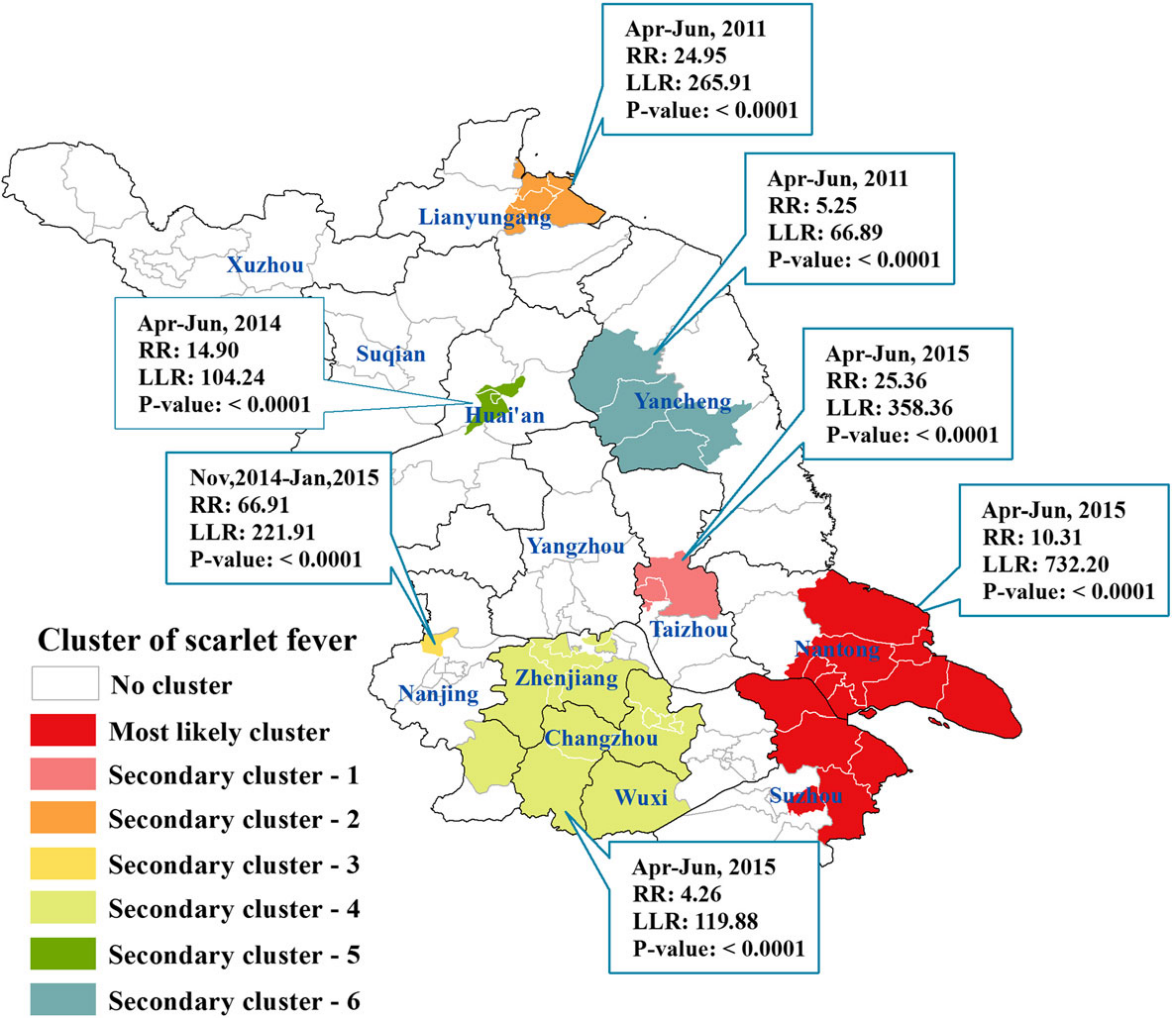
Spatiotemporal clusters of scarlet fever in Jiangsu Province, 2005–2015

Seven clusters were detected in the study period. All of them occurred in the higher incidence peaks, except the third secondary cluster that appeared in the lower incidence peak. The most likely cluster covered 13 counties of Nantong and Suzhou, two southeast cities denoting high endemic regions of scarlet fever in Jiangsu Province. The second secondary cluster covered four counties in Lianyungang city, an endemic region in the north. Notably, two clusters (i.e. the fifth and sixth secondary cluster) were observed in the low endemic regions (Fig. 3).

## Discussion

According to our study, children aged 3–9 years (appropriate ages for kindergarten or primary school in China) were the main victims of scarlet fever, not only accounting for most of the total cases, but also presenting a much higher incidence rate compared to other age groups. This finding was similar to other studies [18, 24–29]. It might be that low immunity and the concentrated population in kindergarten and primary school could promote the spread of scarlet fever. Interestingly, consistent with other researchers’ results [24–29, 37], we also found that boys were more prone to infection. Boys usually have more physical activities than girls and lack of personal hygiene, which increase the chances of exposure to GAS.

During the study period, a seasonal pattern of scarlet fever was observed in Jiangsu Province. Two distinct epidemic peaks occurred each year. The main peak was from March to July, and the smaller peak from November to the following January. Some researchers have argued that seasonal fluctuation of scarlet fever may be attributed to climate conditions [38, 39]. Remarkably, both of the peaks happen to occur in the first or second semester of each school year, respectively. Meanwhile, two incidence troughs were observed during summer (August) and winter (February) holidays. Combining this with the prior results indicating that school aged children were the major group with scarlet fever, we can concluded that kindergarten and primary school should be the focus of surveillance and control for this infectious disease.

Notably, spatial heterogeneity of scarlet fever was detected in Jiangsu Province. The results from maps of the incidence and space-time scan analysis suggested that the incidences in southern regions were relatively higher than in northern regions. Nantong city, Suzhou city and Lianyungang city were high endemic regions. Prevention and control efforts should be strengthened in these areas. Although, the three northwest cities of Xuzhou, Suqian and Huai’an were low endemic regions, some spatiotemporal incidence clusters were detected in these cities. This suggested that local high prevalence areas exist within the low endemic regions. Thus, surveillance, prevention, and control in these regions should be given due importance.

The incidence of scarlet fever presented an increasing trend in recent years in Jiangsu Province. This trend is consistent with other regions of China as well as in other countries, and the reasons remain unknown [1, 12, 14–18, 20]. In this case, some conventional measures would be crucial for prevention and control of this disease. Firstly, teachers and parents need to teach school-age children to wash their hands frequently, this is the best way to prevent the disease. They should also avoid sharing personal items, such as eating utensils and towels [5]. Secondly, schools ought to improve environmental hygiene through disinfection of toys, banisters, and desks, etc. [17]. In addition, more physical activities should be organized for children to enhance their fitness. Meanwhile, public health authorities should more effectively focus on surveillance, prevention, and control of scarlet fever.

In spite of the above findings, the limitations in our study should be considered. Because some mild cases might use home therapies, and some cases with atypical symptoms may be misdiagnosed, the data reported may underestimate the incidence. Moreover, due to a lack of emm types cases, we could not represent the changes of the circulating strains from 2005 to 2015, this is meaningful to interpretation of the increasing incidence of scarlet fever in Jiangsu Province. Hopefully, a genetic molecular study will be conducted in Jiangsu Province.

## Conclusions

In brief, we described the spatiotemporal dynamics of scarlet fever in Jiangsu Province from 2005 to 2015. The prevalence of this disease had an obvious seasonal variation and was relatively highly endemic. School aged children were the major victims, and kindergartens and primary schools should be the focus of surveillance and control for this common disease. The health authorities and policymakers, especially in those endemic areas, should pay close attentions to scarlet fever and make targeted efforts to reduce the incidence rate and maximize the cost-effectiveness of prevention and control programs.

## Abbreviations

GAS: Group A *Streptococcus pyogenes*
GIS: Geographic Information System
LLR: Log Likelihood Ratio
NIDRIS: Nationwide Notifiable Infectious Diseases Reporting Information System
